# Hirayama disease successfully treated by posterior fixation: a case report

**DOI:** 10.1097/MS9.0000000000002076

**Published:** 2024-04-23

**Authors:** Sabik R. Kayastha, Archana Pandey, Abhishek Pandey, Suraj Keshari, Ajit Pandey

**Affiliations:** a Kathmandu University School of Medical Sciences, Dhulikhel Hospital; b Dhulikhel Hospital, Dhulikhel, Nepal

**Keywords:** case report, cervical myelopathy, Hirayama disease, posterior fixation

## Abstract

**Introduction and importance:**

Hirayama disease is a rare form of motor neuron amyotrophy that usually presents with weakness and atrophy of the distal upper extremities in young males. It is believed that it is caused by spinal cord compression during neck flexion because of the widening of the posterior extradural space. This case has been brought to attention due to its extraordinary rarity, serving as an educational tool for medical professionals and to advocate for surgical intervention when deemed necessary.

**Case presentation:**

The authors present a case of a young male in his 20s who was diagnosed with Hirayama disease, had weakness and atrophy in both of his upper limbs, and has been successfully treated by posterior fixation at C4, C5, and C6 with lateral mass screws.

**Clinical discussion:**

The majority of cases stabilize after 2–3 years of progression; therefore, cervical collars are generally sufficient for therapy. However, in certain serious cases with progression even after that time, surgical intervention is an option. Because this is such an uncommon incidence, surgical therapy has not been explored and is controversial.

**Conclusion:**

The use of posterior fixation at C4, C5, and C6 with lateral mass screws as a therapy for Hirayama disease may be regarded as a successful approach.

## Introduction

Highlights

- Dynamic flexion and extension MRI is recommended for the diagnosis of Hirayama disease especially in young males with unexplained motor neuropathy.
- In the majority of cases, cervical collars are adequate for treatment, but not in all cases of Hirayama disease.
- Posterior fixation may be a useful surgical strategy for treating Hirayama disease.
- This case report highlights the importance of considering surgical treatment in cases where conservative measures fail to alleviate symptoms or halt disease progression.

Hirayama disease, which is also known as monomelic amyotrophy, juvenile asymmetric segmental spinal muscular atrophy [JASSMA], and juvenile muscular atrophy of the distal upper extremity is an extremely rare disease^1^. The condition usually affects men in their 20s^2^. It is a benign, self-limiting cervical myelopathy in the distribution of the muscles that are innervated by spinal segments C7, C8, and T1^3^. Hirayama first reported it as affecting just one upper extremity, but contrary to previous observations, asymmetric bilateral involvement was detected in the majority of cases, giving rise to the term JASSMA^4^. Typically, patients develop unilateral or asymmetric atrophy of the hand and forearm, sparing the brachioradialis, which gives rise to the distinctive look of oblique amyotrophy involving the C7, C8, and T1 myotomes^1^. There is a forward displacement of the spinal cord and compression of the cervical cord by enlargement of the posterior extradural space when the neck is flexed^5^. Circulatory disruption was thought to be the pathophysiological process since there were less anterior horn cells visible under a microscope^6^.

Here, we report a case of a male in his 20s who presented to our tertiary care hospital with muscular atrophy in bilateral distal upper extremities. The case has been documented because of its rarity, educational value, and to advocate a surgical approach when necessary. This case report has been reported in line with Surgical CAse REport (SCARE) 2023 Criteria^7^.

### Patient information

A 22-year-old male presented with progressive weakness and wasting between the thumb and index finger of his left hand. He has a history of similar symptoms on his right hand. He had trouble writing and picking up small objects. Weakness in both arms gradually progressed over 4 years before he presented to our hospital. He also complaints of tremors in bilateral hands. There is no history of trauma, tingling, or numbness. The patient’s medical and surgical history is unremarkable. The patient reports no prior use of medications and has no known allergies. No familial occurrences of similar illnesses were identified during the patient’s family history assessment. Additionally, the patient denies any history of smoking, alcohol intake, or recreational drug use.

### Clinical findings

**Figure 1:**
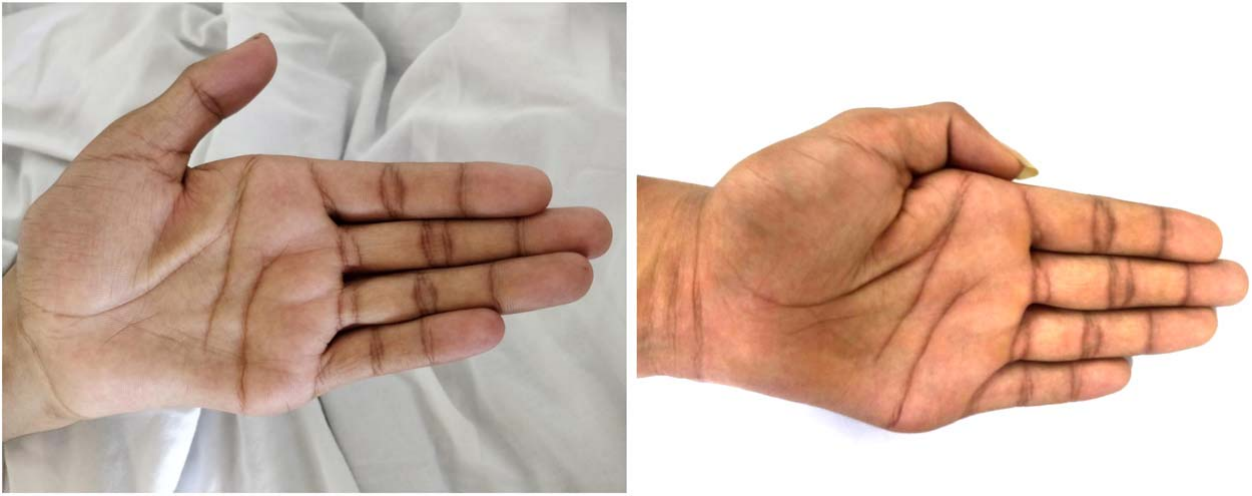
Mild atrophic changes were observed in the thenar and hypothenar eminences during the preoperative (left) and postoperative (right) periods, with more marked changes noted during the preoperative period.

On examination, there was wasting over the palmar aspect of bilateral hands between the thumb and index finger as observed in Figure 1. Tremors were noticeable in both hands. Tone and sensory conditions were both normal. Motor function was found to be normal (5/5) except at the level of C8 (3/5) on the left side. Reflexes were brisk with bilateral down going Babinski. Deep tendon reflexes were normal bilaterally in the upper limbs. Sensation was intact bilaterally. The plantar reflex was normal. Systemic examinations, including the gastrointestinal, respiratory, and cardiovascular systems, detected no abnormalities.

### Diagnostic assessment and interpretation

A nerve conduction study revealed a decreased amplitude of the left ulnar nerve compound muscle action potential (CMAP) and normal amplitude on the opposite side, with normal sensory nerve action potentials (SNAP) signifying left ulnar motor axonal neuropathy. This finding effectively ruled out amyotrophic lateral sclerosis and cervical spondylotic amyotrophy. Complete blood count, kidney function test, and liver function tests were all within normal ranges. Serological testing was negative, excluding the possibility of HIV.

**Figure 2:**
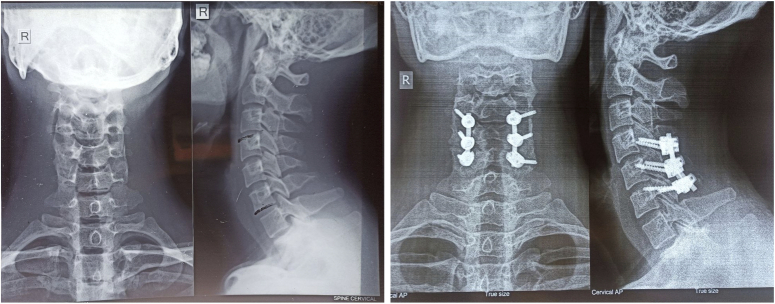
Normal cervical lordosis noted in both preoperative (left) and postoperative (right) images with lateral mass screws in C4, C5, and C6 vertebrae with connecting rods observed in postoperative image (right).

**Figure 3:**
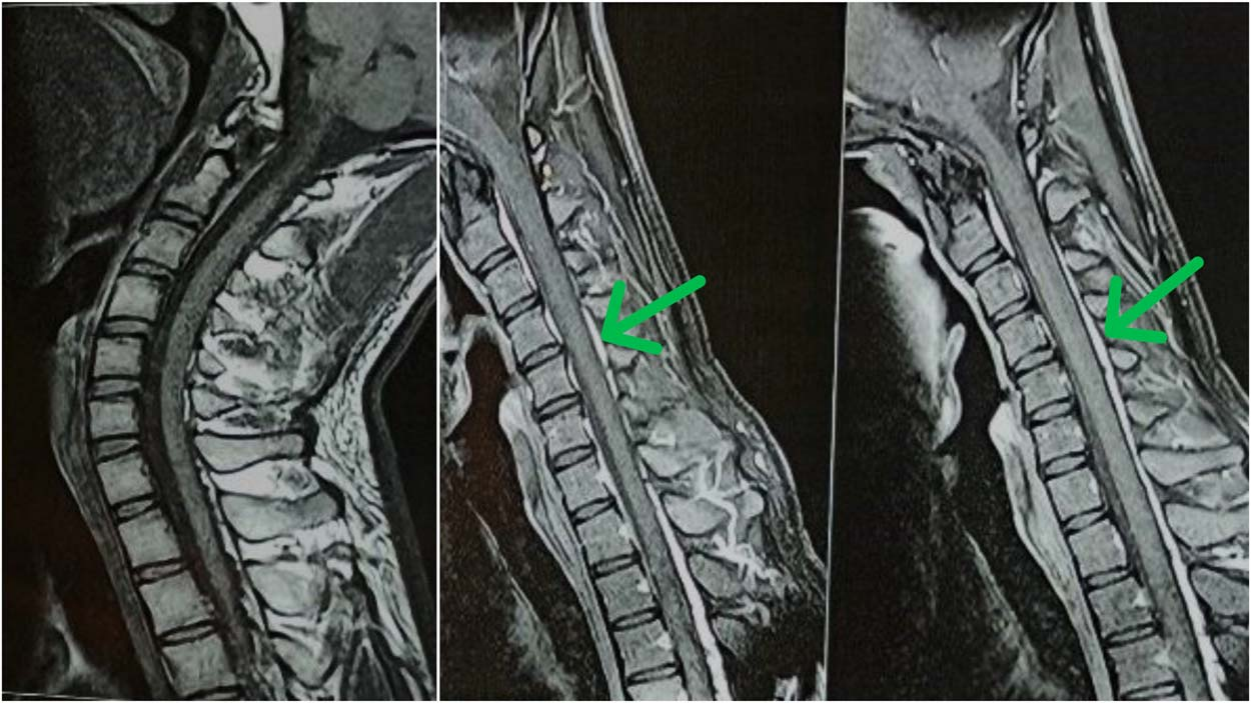
MRI of C-Spine (Left: Extension; Middle and Right: Flexion) shows anterior displacement of the dura with prominent epidural space from C4 to C6 level in flexion sequence with enhancement on post contrast study, no signal changes in spinal cord.

Radiographs ruled out any osteophytes or bony deformities (Fig. 2). MRI, axial, and sagittal T1 and T2 weighted images in flexion and extension showed anterior displacement of the posterior dural wall with prominent homogeneously enhancing epidural space in flexion sequences from C4 to C6 level, causing compression of the adjacent spinal cord and leading to its focal cord atrophy at the same level. However, no signal intensity was seen in the spinal cord (Fig. 3).

Based on the history, muscle involvement, nerve conduction testing, and MRI results, Hirayama disease was diagnosed. The patient was initially advised to wear a cervical collar to prevent the flexion of his neck and further compression of the spinal cord. Even after using a cervical collar for about 6–7 months, there was no improvement in symptoms. Thus, the patient was scheduled for surgery. We opted for posterior fixation as the preferred surgical approach for our patient due to the presence of dynamic instability and the surgeon’s preference. This decision was made after careful consideration of the patient’s condition, emphasizing the need for stabilization of the cervical spine to address the underlying instability effectively.

### Intervention

The patient was placed in the prone position, Mayfield head clamp was applied, and cervical lordosis was maintained, which was confirmed on fluoroscopy. After shaving, his skin was painted with betadine, and draping was done. Under general anesthesia, a midline posterior approach incision of about 10 cm was made, running from C3 to C7, and subcutaneous tissues and fascia were dissected up to the lamina with lifting of the paraspinal muscles subperiosteally. Then the lateral mass was exposed from C4 to C6. Lateral mass screws were placed bilaterally over C4 (3.5×18 mm), C5 (3.5×16 mm), and C6 (3.5×20 mm) vertebral levels, and then joined with two connecting rods after contouring the rods to maintain cervical lordosis. The C-arm was used to confirm screw position, screw length, and neck alignment. Along with administering vancomycin powder, the standard closure was performed in layers over a negative suction. Following surgery, the patient was given a Philadelphia brace to keep his neck in that position for a month.

### Follow-up and outcomes

After a total of 5 days in the hospital, the patient was discharged on tablet cefuroxime 500 mg twice daily for 7 days with analgesics. He was instructed to have wound dressing performed on alternate days. On the third postoperative day, the wound drain was removed. The sutures were removed on the 10th postoperative day. Physical therapy commenced 3 weeks postsurgery and continued for a duration of 4 months, during which the intensity and duration of the sessions were gradually augmented based on the recommendations provided by the physiotherapist. The patient had a good recovery. There were no intraoperative or postoperative complications. On 1-month follow-up, his motor grade slightly improved. At 1-year postoperatively, there was a modest reversal of muscular wasting and improvement in the wasting of his upper extremities. Although the symptoms did not completely relieve, the patient reported a significant improvement in his daily activities. A 1-year follow-up radiograph revealed screws at the posterior elements of C4 to C6 vertebral levels (Fig. 2). This case demonstrates the patient’s remarkable adherence and tolerance postoperatively. Through consistent medication use, attending follow-ups in outpatient department, and proper wound care, he achieved a smooth recovery, emphasizing the importance of patient commitment for successful outcomes.

We plan to monitor the patient on a regular basis, with follow-ups every 6 months for the first 3 years and then once a year thereafter. Each follow-up will include a clinical examination and radiographs to assess the patient’s progress.

## Discussion

Our case report describes a rare case of a young male who developed progressive hand weakness and wasting. He was diagnosed with Hirayama disease, which was confirmed by MRI findings showing anterior dural displacement with a prominent epidural space from C4 to C6 levels in flexion sequences, as well as by clinical examination and nerve conduction studies. Despite initial conservative treatment, surgical intervention with posterior cervical fixation at C4, C5, and C6 using lateral mass screws resulted in improved motor function and muscular wasting reversal at 1-year follow-up. This suggests that surgical treatment should be considered in cases where conservative measures fail to alleviate symptoms or halt disease progression. The case highlights the efficacy of surgery in managing Hirayama disease and emphasizes the importance of regular follow-up for monitoring long-term outcomes.

Hirayama disease was described by Hirayama in 1959 with ~200 cases reported in literature^8^. It is a sporadic juvenile muscular atrophy of the distal upper limbs. The predisposing factors include male sex, with a male-to-female ratio of 7:1, an age group of 14–38 years, and Asian descent.^9^. The characteristic clinical features as originally described by Hirayama include (a) weakness and wasting predominantly in C7, C8, and T1 myotomes in one upper limb or asymmetrically in both upper limbs, (b) insidious onset in teens or early 20s, (c) progression for 1–3 years followed by arrest of disease or relatively benign course, (d) irregular coarse tremors (minipolymyoclonus) in the fingers of the affected hand(s), (e) mild transient worsening of symptoms on exposure to cold, (f) electromyography evidence of chronic denervation in the clinically or sub clinically affected muscles, and (g) absence of substantial sensory loss/reflex abnormalities, cranial nerve, pyramidal tract in the lower limb, sphincter, or cerebellar deficits^10^. C5–C7 segmental myotomes are commonly involved in this disease in the population of western countries, whereas C7–T1 segments are commonly involved in Asian countries^11^. All of these points were fulfilled by our case, which helped make the diagnosis.

During neck extension, the dura mater becomes slack and folds transversely. As the neck moves from extension to flexion, the dura becomes tighter, because the length of the cervical canal increases. From T1 to the top of the atlas, there is a 1.5 cm variation in length at the anterior wall and a 5 cm difference at the posterior wall between extension and flexion^12^. In Hirayama disease, the Dural canal is no longer slack in extension because of an imbalance in the growth the vertebrae and the dura mater. Therefore, a tight Dural canal is formed, which cannot compensate for the increased length of the posterior wall during flexion. This results in the posterior dural wall shifting anteriorly, compressing the cord as a result. Microcirculatory abnormalities in the territory of the anterior spinal artery or the anterior portion of the spinal cord may result from this compression. The chronic circulatory disturbance resulting from repeated or sustained flexion of the neck may produce necrosis of the anterior horns, which are most vulnerable to ischemia^13^.

Cervical radiographs often reveal no changes, as was the case here. MRI of the neck in flexion often indicates anterior displacement of the posterior dural sac as well as a crescent mass in the posterior epidural space of the lower cervical canal^14^. The nerve conduction study showed a decrease in the amplitude of the left ulnar nerve CMAP, which was consistent with the findings of Hirayama disease and ruled out amyotrophic lateral sclerosis with a higher CMAP and cervical spondylotic amyotrophy with an indifferent value from the normal range.^15^

Other conditions, like demyelinating polyneuropathy in HIV, congenital neuropathies, postpolio syndrome, and syringomyelia should be ruled out by history and appropriate clinical investigation. Diagnosis is made based on clinical and diagnostic findings, and MRI and EMG are often necessary tests. Monomelic amyotrophy is a self-limiting condition without definitive treatment. Cervical collars can be used to prevent neck flexion, compression, and mitigate symptom exacerbation. Apart from permanent cervical fixation, posterior cervical decompression with coagulation of epidural venous plexus is a technique that seems to be effective^16^. Physiotherapy can help prevent complications associated with immobility, such as joint stiffness and muscle wasting^17^.

Surgical intervention remains an option for individuals who do not respond to a cervical collar. However, since different surgical techniques can be used, outcomes are unpredictable, and most cases tend to stabilize naturally after 2–3 years of progression, surgical treatment is controversial^18^. Surgical procedures have included multilayer anterior corpectomies, multilevel discectomies, decompressive laminectomies, Dural grafting, ‘tenting’ Dural surgery, and a variety of laminoplasties. The current standard of care generally consists of anterior and/or posterior fusions together associated with decompression or removal of midline bone elements. In a study involving five patients post spinal fixation, all five patients had clinical improvement, suggesting multilevel spinal instability may be the defining cause of Hirayama disease^19^. Similarly, other studies relating to individuals who underwent surgery reported improved or stable muscular strength^19–21^. In this case, both muscle strength and wasting improved after a 12-month follow-up.

## Strengths and limitations

The positive response observed during the 12-month follow-up highlights the effectiveness of posterior fixation in managing the condition. Nevertheless, the single-case nature of the report constrains its generalizability. The lack of a comparative analysis, especially concerning surgery, along with the short-term focus, presents challenges in achieving a comprehensive understanding.

## Conclusion

The use of posterior fixation at C4, C5, and C6 with lateral mass screw as a therapy for Hirayama disease may be regarded as a successful approach. Our patient’s symptoms of weakness and wasting improved after surgery, despite no improvement during conservative management with a cervical collar. This suggests that surgical treatment should be considered in cases where conservative measures fail to alleviate symptoms or halt disease progression. As a result, we believe that posterior fixation may be an effective option. We also want to highlight the importance of a dynamic flexion and extension MRI for the diagnosis of Hirayama disease, particularly in young men with unexplained motor neuropathy.

## Ethical approval

Ethical approval for this study is not required by the Kathmandu University School of Medical Sciences-Institutional Review Committee (KUSMS-IRC).

## Consent

Written informed consent was obtained from the patient for publication of this case report and accompanying images. A copy of the written consent is available for review by the Editor-in-Chief of this journal on request.

## Sources of funding

Funding was not received for this study.

## Author contribution

S.R.K.: concept, data collection, manuscript preparation, edit and review, and guarantor; A.P.: manuscript preparation, obtaining consent from the patient, and edit and review; A.P., A.P., and S.K.: manuscript preparation and edit and review.

## Conflicts of interest disclosure

All authors declare that they have no conflicts of interest.

## Research registration unique identifying number (UIN)

1. Name of the registry: not applicable.
2. Unique identifying number or registration ID: not applicable.
3. Hyperlink to your specific registration (must be publicly accessible and will be checked): not applicable.

## Guarantor

Dr Sabik Raj Kayastha MS Orthopedics, Fellowship in Spine Surgery, Department of Orthopedics, Dhulikhel Hospital Dhulikhel, Kavre.

## Data availability statement

Data sharing is not applicable to this article.

## Provenance and peer review

Not commissioned, externally peer-reviewed.
